# *Sfrp5* identifies murine cardiac progenitors for all myocardial structures except for the right ventricle

**DOI:** 10.1038/ncomms14664

**Published:** 2017-03-13

**Authors:** Masayuki Fujii, Akane Sakaguchi, Ryo Kamata, Masataka Nagao, Yutaka Kikuchi, Silvia M. Evans, Masao Yoshizumi, Akihiko Shimono, Yumiko Saga, Hiroki Kokubo

**Affiliations:** 1 Department of Cardiovascular Physiology and Medicine, Graduate School of Biomedical and Health Sciences, Hiroshima University, 1-2-3 Kasumi, Minamiku, Hiroshima 734-8551, Japan; 2 Department of Mammalian Development, National Institute of Genetics, Mishima 411-8540 Japan; 3 The Graduate University for Advanced Studies (SOKENDAI), Hayama, Kanagawa 240-0193, Japan; 4 Department of Forensic Medicine, Graduate School of Biomedical and Health Sciences, Hiroshima University, 1-2-3 Kasumi, Minamiku, Hiroshima 734-8551, Japan; 5 Department of Biological Science, Graduate School of Science, Hiroshima University, 1-3-2 Kagamiyama, Higashi-hiroshima 739-8511, Japan; 6 Department of Medicine, School of Medicine UC San Diego, 9500 Gilman Drive, La Jolla, California 92093, USA; 7 Center for Developmental Biology (CDB), RIKEN Kobe, 2-2-3 Minatojima-minamimachi, Chuou-ku, Kobe 650-0047, Japan; 8 Department of Biological Sciences, Graduate School of Science, The University of Tokyo, Hongo 7-3-1, Bunkyo-ku, Tokyo 113-0033, Japan; 9 Present address: Bristol-Myers K.K. 6-5-1 Nishi-Shinjuku, Shinjuku-ku, Tokyo 163-1328, Japan

## Abstract

Upon acquirement of pulmonary circulation, the ancestral heart may have been remodelled coincidently with, or accompanied by, the production and rearrangement of progenitor cells. However, the progenitor populations that give rise to the left ventricle (LV) and sinus venosus (SV) are still ambiguous. Here we show that the expression of Secreted frizzled-related protein *Sfrp5* in the mouse identifies common progenitors for the outflow tract (OFT), LV, atrium and SV but not the right ventricle (RV). *Sfrp5* expression begins at the lateral sides of the cardiac crescent, excluding early differentiating regions, and continues in the venous pole, which gives rise to the SV. Lineage-tracing analysis revealed that descendants of *Sfrp5*-expressing cells at E7.5 contribute not only to the SV but also to the LV, atria and OFT and are found also in the dorsal splanchnic mesoderm accompanied by the expression of the secondary heart field marker, *Islet1*. These findings provide insight into the arrangement of cardiac progenitors for systemic circulation.

## Abstract (web summary)

It is unclear which progenitors define different regions of the heart. Here, the authors find Secreted frizzled-related protein 5 is expressed in murine progenitor cells for the outflow tract, first heart field, and sinus venosus, but not the right ventricle, and Wnt inhibition prevents progenitor proliferation.

Mammalian cardiac development begins with the cardiac crescent, which is composed of a bilateral cardiogenic splanchnic mesoderm, and the progressive differentiation of progenitors into cardiomyocytes to form the primary heart tube. During the successive development of the four-chambered heart, two independent pulmonary and systemic circulatory systems are established. In this process, the production and rearrangement of progenitor cells may account for remodelling of the ancestral heart into these two separate systems. Recent findings pertaining to another cardiac lineage called the secondary heart field (SHF) modified the classical view of cardiac development that the myocardium was derived from a single source. The SHF emerges in the splanchnic mesoderm to supply cells at both the arterial and venous poles of the heart tube, including the right ventricle (RV), most of the atria and outflow tract (OFT)^1^,^2^,^3^. However, the progenitor population that forms the first heart field (FHF), which gives rise to the left ventricle (LV) and the rest of the atria, and the sinus venosus (SV) are still ambiguous.

SHF progenitor cells, which transiently express *Islet1* (*Isl1*), have been shown to express cardiac markers, including *Fibroblast growth factor 10* (*Fgf10*), *Myocyte enhancer factor 2c* (*Mef2c*), *T-box transcription factor 1* (*Tbx-1*), as well as other markers, and contribute to the RV, the OFT and most of the atria^1^,^2^,^4^,^5^,^6^,^7^,^8^,^9^. FHF progenitor cells have yet to be defined because the FHF lineage, which subsequently gives rise to the LV and a subset of the atria, is recognized only after the expression of differentiation markers such as *Myosin light chain 2a* (*Mlc2a*) and *NK-2 transcription factor related, locus 5 (Nkx2-5)* in the cardiac crescent^3^,^10^,^11^.

The SV is a cavity formed by a thin cardiac muscular wall, which drains blood from systemic veins and the coronary sinus into the right atrium, and gives rise to the sinoatrial node at the joint to the superior cardinal vein during embryogenesis. The origin of the SV has been previously addressed and mapped to the lateral rim of the heart fields in chickens by dye injection experiments^12^. Recently, the cell population in the lateral rim of the heart fields, which was defined by the expression of *mutG5/hsp68/LacZ* in mice, was found to contribute to the SV^13^. The venous pole was reported to be the precursor of the SV, which is marked by the expression of *Tbx18* from E8.25 (refs 13, 14). Lineage-tracing experiments with *Tbx18-Cre* mice revealed that *Tbx18-*expressing cells form the SV and proepicardium^15^,^16^. Thus the origin of the SV has been suggested to be in the caudolateral regions of the cardiac crescent, although its relation to the FHF or SHF remains unclear.

Wnt signalling is one of the most important regulatory pathways in vertebrate cardiogenesis^17^,^18^,^19^,^20^,^21^. Wnt antagonism by Dkk1 or Crescent induces heart formation in vertebrates^22^,^23^,^24^, and Dkk1 is required for head induction in mammals^25^. Secreted frizzled-related protein (Sfrp) 1 and Sfrp2, which are also Wnt inhibitors, are expressed in the heart and are involved in cardiac tissue repair^26^,^27^, while the expression pattern and function of another subfamily member, Sfrp5, during cardiac development are unknown.

In this report, we show that *Sfrp5* expression is detected in lateral and caudal regions of the cardiac crescent, is expressed in cells distinct from those positive for markers of differentiated cardiomyocytes and the SHF and continues to be expressed in the developing SV. However, descendants of *Sfrp5*-expressing cells notably contribute to cardiomyocytes in all chambers of the heart, except for the RV. These observations suggest that *Sfrp5*-expressing cells in the lateral region of the cardiac crescent are progenitor cells for the OFT, the LV, the left and right atria and the SV. Hyperplasia in the inflow tract (IFT) was observed in *Sfrp1/2/5* triple knockout (TKO) mouse embryos, suggesting that Sfrp5 regulates the proliferation of cardiac progenitor cells in the venous pole.

## Results

### *Sfrp5* is expressed in progenitor cells of the SV and epicardium

**Figure 1:**
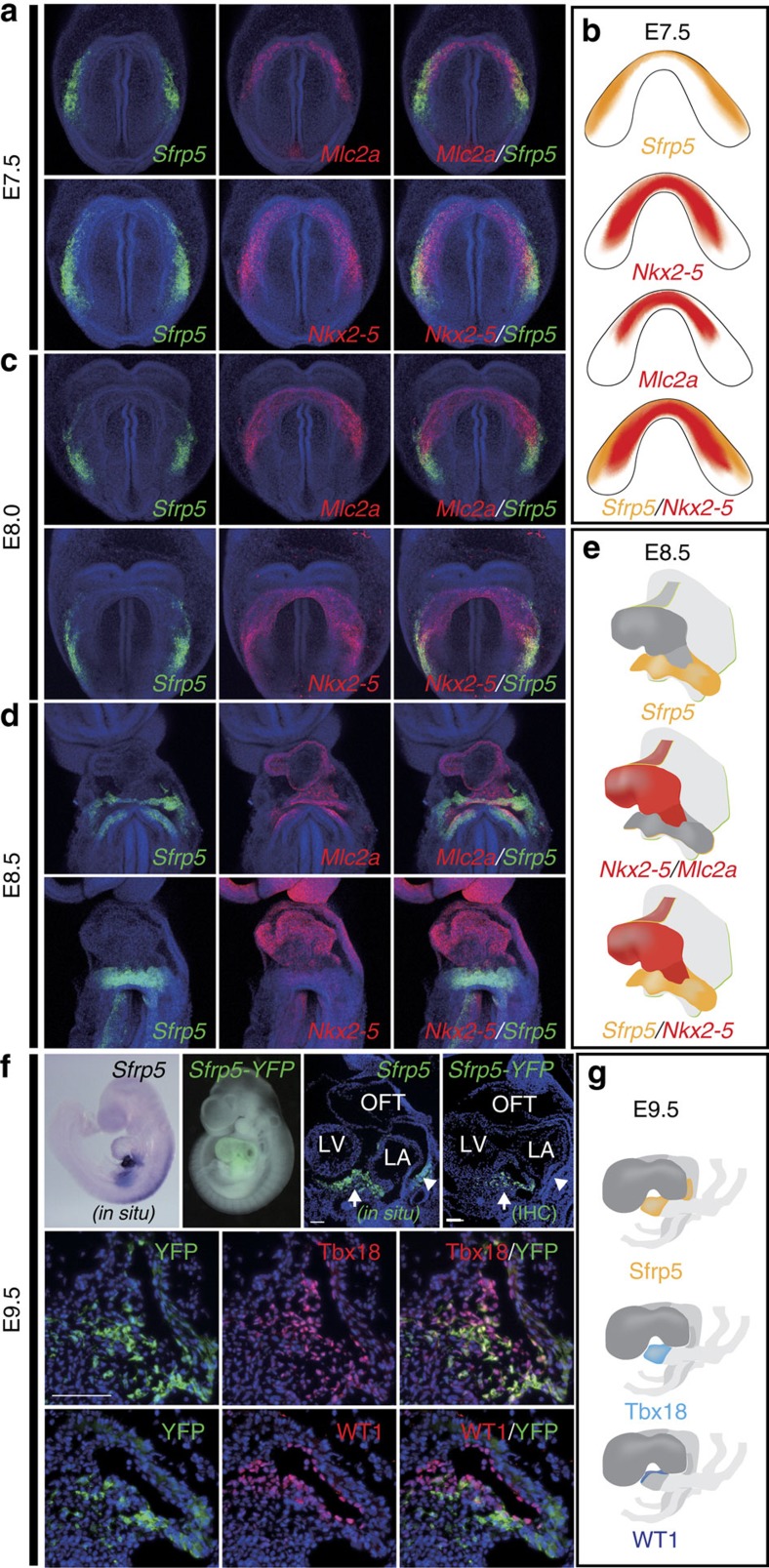
*Sfrp5* is expressed in the lateral cardiac crescent and subsequently in the SV. (**a**,**c**,**d**) Double fluorescent whole-mount ISH of mouse embryos at E7.5 (**a**), E8.0 (**c**) and E8.5 (**d**) for detection of *Sfrp5* (green) using *Mlc2a* or *Nkx2-5* (red) as indicated in each panel. (**b**,**e**,**g**) Schematic drawings of gene expression (**b**,**e**) and protein distribution (**g**). (**f**) Whole-mount ISH (*in situ*), YFP image merged with bright field image (*Sfrp5-YFP*), section *in situ* with probe for *Sfrp5* and single or double immunohistochemistry (IHC) of *Sfrp5-venusYFP* KI embryos using anti-GFP antibodies with anti-Tbx18 or anti-WT1 antibodies at E9.5, as indicated in each panel. Scale bars=Scale bars, 50 μm.

Wnt signalling is required for myocardial induction and is implicated in several steps of heart development^17^,^18^,^19^,^20^,^21^. The *Sfrp* subfamily genes (*Sfrp1*, *2* and *5*) are known to encode secreted decoy receptors for Wnt signalling^28^ and to participate in the maintenance of adult cardiac function^26^,^29^. Among the three *Sfrp* genes, we found that *Sfrp5* was specifically expressed in a particular part of the cardiac crescent at E7.5. *Sfrp5* was detected in regions lateral and caudal to the region of *Mlc2a* and *Nkx2-5* expression, which marks differentiating cardiomyocytes (Fig. 1a,b). The expression area of *Sfrp5* was clearly distinct from those of *Mlc2a* and *Nkx2-5* (Supplementary Fig. 1). During the heart tube looping stage (E8.0–E8.5), the expression of *Sfrp5* was observed continuously in the venous pole of the heart (Fig. 1c–e), the pattern of which was distinct from *Mlc2a* and *Nkx2-5*, indicating that the *Sfrp5*-expressing area excludes differentiating cardiomyocytes. At E9.5, *Sfrp5* expression was confined to the ventral venous pole, which is known to contain cardiac progenitors for the SV as well as the epicardium, suggesting that *Sfrp5* might be expressed in such progenitors. To investigate this possibility, we performed double fluorescent immunohistochemistry using Tbx18 and WT1, specific markers for the SV and epicardium, respectively. To monitor the Sfrp5 expression, we used *Sfrp5-venusYFP* embryos. At E9.5, yellow fluorescent protein (YFP) was detected strongly in the ventral side and weakly in the dorsal side of the venous pole, similar to the pattern of *Sfrp5* expression (Fig. 1f). The YFP signal highly overlapped with Tbx18 staining and moderately with WT1 staining (Fig. 1f,g), suggesting that *Sfrp5* is expressed in progenitor cells of the SV and the epicardium.

Cardiomyocytes in the SV show a unique gene expression profile, that is, TroponinT (TnT)- and Hcn4-positive but Nkx2-5-negative. This differs from the Nkx2-5- and TnT-positive chamber myocardium^30^. To further examine the expression profile of *Sfrp5-venusYFP-*expressing cells, we conducted marker expression analysis. At E9.5, *Sfrp5-venusYFP*-expressing cells were negative for Nkx2-5, TnT and Hcn4 (Supplementary Fig. 2a), whereas, at E10.5, these cells expressed TnT and Hcn4 but not Nkx2-5 (Supplementary Fig. 2b). These results indicate that *Sfrp5*-expressing cells in the ventral venous pole at E9.5 represent progenitors for the SV and that they differentiate into cardiomyocytes in the SV at E10.5. Thus the *Sfrp5*-expressing area at E7.5 may be the precursor of the SV.

### *Sfrp5* is expressed in progenitors of cardiac components except for RV

**Figure 2:**
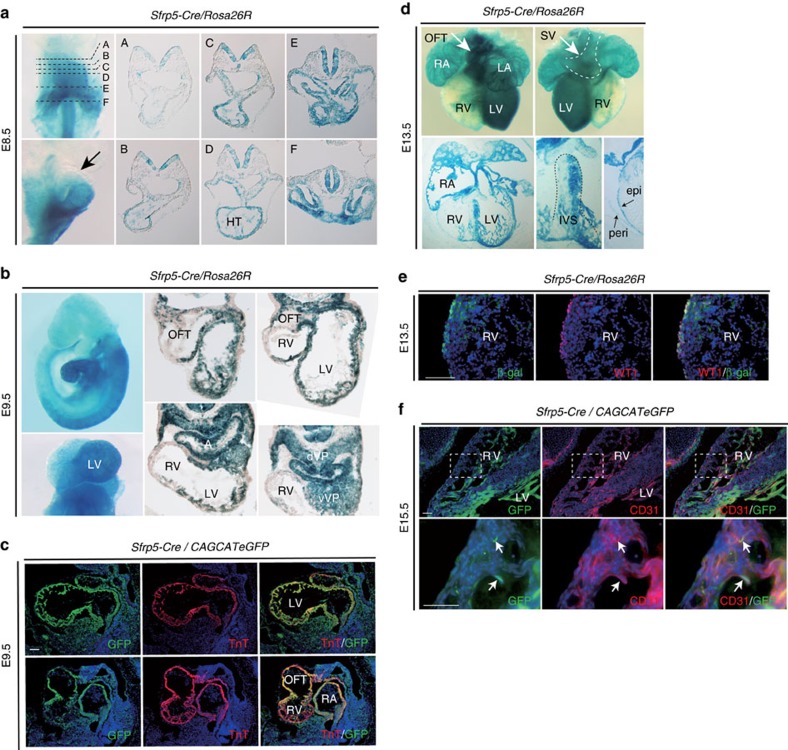
Descendants of *Sfrp5*-expressing cells contribute to the SV and all chamber myocardium except for the RV. (**a**,**b**,**d**) Whole-mount and section LacZ staining of *Sfrp5-Cre/Rosa26R* embryos at E8.5 (**a**), E9.5 (**b**) and E13.5 (**d**). HT: heart tube; dVP: dorsal venous pole; vVP: ventral venous pole; epi: epicardium; peri: pericardium, IVS; interventricular septum. (**c**,**e**,**f**) Double immunohistochemistry of *Sfrp5*-*Cre*/*CAG-floxed-CAT-eGFP* (**c**,**f**) or *Sfrp5*-*Cre*/*Rosa26R* (**e**) embryos at E 9.5 (**c**), E 13.5 (**e**) and E15.5 (**f**), using anti-GFP or anti-β-gal (green) antibodies with anti-TnT, anti-Isl1, anti-WT1 or anti-CD31 antibodies (red). Rectangles in upper panels (**f**) are magnified in lower panels. Arrows indicate cells co-expressing YFP and CD31 (**f**). Scale bars=50 μm.

We next determined whether progenitors of the SV correspond to *Sfrp5*-expressing cells in the cardiac crescent. To test this possibility, we generated a *Sfrp5*-*Cre* line (Supplementary Fig. 3) and investigated the lineages of *Sfrp5*-expressing cells by crossing this line with *Rosa26R* or *CAG-floxed-CAT-eGFP* reporter mice^31^. At E8.5, β-galactosidase (LacZ) staining was unexpectedly observed in the entire heart tube, except for the most rostral portion (Fig. 2a). At E9.5, LacZ staining was observed in the OFT, LV, atria and venous pole but not in the RV (Fig. 2b). Labelled cells overlapped with TnT in the OFT, LV and atria, suggesting that descendants of *Sfrp5*-expressing cells differentiated into chamber myocardium in addition to the SV (Fig. 2c). At E13.5, LacZ-stained cells mostly occupied not only the LV but were also found in the WT1-expressing pericardium (Fig. 2d,e) and the cluster of differentiation 31 (CD31)- or platelet endothelial cell adhesion molecule-expressing endocardium (Fig. 2f). Labelled cells were found in part of the interventricular septum and rarely in the RV (Fig. 2b,d) and those that were found in the RV tended to localize in the trabecular myocardium (Fig. 2f). These observations suggest that *Sfrp5* is expressed in progenitors of the SV, pericardium, epicardium and endocardium, as well as in all chamber myocardium except for the RV. Given that *Isl1* lineages contribute to all myocardium except for the LV^32^,^33^, the fate of *Isl1* lineages to the RV or *Sfrp5* lineages to the LV may be exclusively determined during the earliest stage of cardiogenesis.

**Figure 3:**
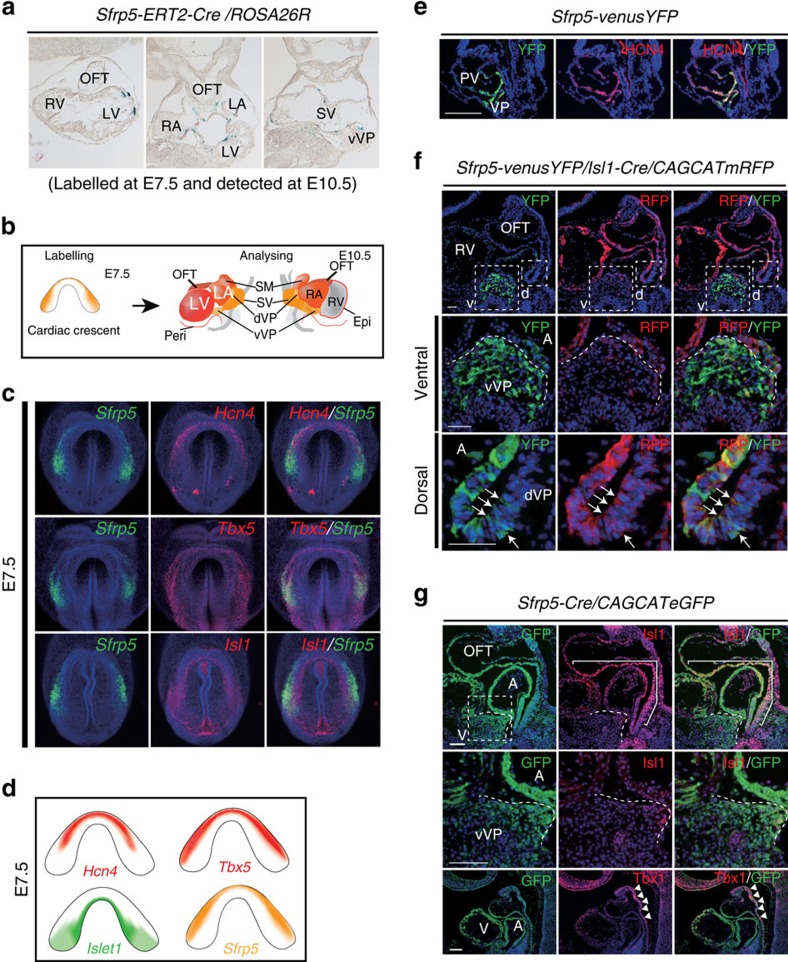
*Sfrp5-*expressing cells at E7.5 contribute to the SV and all chamber myocardium except for the RV. (**a**) Representative photographs of LacZ-stained sections from *Sfrp5-ERT2Cre*/*Rosa-lacZ* embryos at E10.5, labelled by tamoxifen at E7.5. (**b**) Schematic drawing for the expression of *Sfrp5* (orange) and the cardiac contribution of its lineages (red) when cells were labelled at E7.5 and analysed at E10.5. (**c**) Double fluorescent whole-mount ISH of mouse embryos at E7.5 for detection of *Sfrp5* (green) and *Hcn4*, *Tbx5* or *Isl1* (red), as indicated in each panel. (**d**) Schematic drawings of gene expression of *Sfrp5*, *Isl1*, *Hcn4* and *Tbx5*. (**e**) Double immunohistochemistry of *Sfrp5-venusYFP* at E8.5 using anti-GFP (green) and anti-HCN4 (red) antibodies. Co-expression with HCN4 was found in the VP. Weak expression of YFP also co-distributed with HCN4-positive cells in the IFT and part of the primitive ventricle (PV). (**f**,**g**) Double immunohistochemistry of *Sfrp5-venusYFP* (**e**), *Sfrp5-venusYFP/Isl1*-*Cre*/*CAG-(floxed)-CAT-mRFP* (**f**) or *Sfrp5*-*Cre*/*CAG-(floxed)-CAT-eGFP* (**g**) embryos at E9.5 using anti-GFP (green) antibodies and anti-HCN4, anti-RFP, anti*-*Isl1 or anti-Tbx1 (red) antibodies. Rectangles in upper panels are magnified in lower panels (v: ventral; d: dorsal). Co-expression with Isl1 was detected (arrows in panel (**f**) and parenthesis in panel (**g**)). Boundaries for the VP from the atrium are indicated by white dotted lines (**f**,**g**). Signal for Tbx1, but not GFP, was observed in the anterior splanchnic mesoderm and anterior OFT (arrowheads in panel (**g**)). A, atrium.

Since *Sfrp5* expression is confined to the venous pole of the heart at E8.5, which does not include chamber myocardium, we speculated that cells expressing *Sfrp5* prior to E8.5 might include progenitors of the FHF. To evaluate the fate of *Sfrp5*-expressing cells at specific developmental stages, we generated a tamoxifen-inducible (*Estrogen Receptor T2* (*ERT2*)) *Cre* mouse line (Supplementary Fig. 3). After crossing *Sfrp5*-*ERT2Cre* mice with reporter mice, *Sfrp5*-expressing cells were labelled at E7.5 via tamoxifen injection and analysed at E10.5. LacZ staining revealed that cells labelled at E7.5 were found in the OFT, LV, atria, SV and venous pole but not in the RV (Fig. 3a,b). Interestingly, labelled cells were still found in the atria at E8.5 and E9.5 (Supplementary Fig. 4), indicating that *Sfrp5*-expressing cells at the venous pole may supply cardiomyocytes for the IFT. These data suggest that *Sfrp5*-expressing cells at E7.5 include common progenitors for the SV, OFT and all chamber myocardium except for the RV. There is therefore a strong possibility that *Sfrp5* expression marks progenitors of the FHF.

### *Sfrp5* derivatives contribute to the FHF and part of the SHF lineages

To test this possibility, the expression of *Sfrp5* was compared with that of the FHF marker genes, *Hcn4* and *Tbx5*. *Hcn4* is expressed in the prospective LV at E7.5, with expression shifting towards the SV^30^,^34^. *Tbx5* is expressed in the FHF lineage at E6.5–E7.5 (refs 35, 36, 37). The expression of *Hcn4* was observed in the anteromedial region compared with *Sfrp5*, while region marked by *Tbx5* overlapped with and extended anteriorly from that marked by *Sfrp5* (Fig. 3c,d). Since the expression of *Hcn4* was restricted to the anterior portion of the *Tbx5* expression area, we concluded that *Hcn4* might mark only the early differentiated FHF lineage. Conversely, *Tbx5* marks both undifferentiated and differentiating FHF progenitors, among which *Sfrp5* expression is restricted to undifferentiated FHF progenitors existing in the lateral sides of the cardiac crescent. Moreover, cells weakly expressing YFP in the primitive ventricle and IFT were co-distributed with HCN4 in the *Sfrp5*-*venusYFP* mouse embryo at E8.5 (Fig. 3e), suggesting that descendants of *Sfrp5*-expressing cells contribute to the FHF lineage.

Lineage analysis also revealed that *Sfrp5*-expressing cells at E7.5 contributed to the OFT and atria, indicating that these cells also include progenitors of the SHF. However, at E7.5, the expression pattern of *Sfrp5* was distinct from that of *Isl1*, an SHF marker, although some overlap was found in the expression in the caudal region (Fig. 3c,d). At E9.5, *Isl1* transcripts and protein were expressed in the splanchnic mesoderm, including the dorsal venous pole, but were rarely expressed in the ventral venous pole, which contains *Sfrp5*-expressing cells (Supplementary Figs 5 and 6). To clarify whether the expression of *Sfrp5* precedes that of *Isl1* in the splanchnic mesoderm, we conducted lineage-tracing analysis using *Isl1-Cre* and *CAG-floxed-CAT-mRFP reporter/Sfrp5-venusYFP* mice. We found that the YFP signal in the ventral venous pole rarely overlapped with derivatives of *Isl1*-expressing cells (Fig. 3f). In *Sfrp5-Cre*/*CAG-floxed-CAT-eGFP-reporter* mice, however, GFP-labelled cells expressed *Isl1* and *Fgf10* in the dorsal splanchnic mesoderm and part of the OFT (Fig. 3g and Supplementary Fig. 7). These findings suggest that derivatives of *Sfrp5*-expressing cells also contributed to part of the SHF lineage, which may give rise to the OFT and atria. Notably, these GFP-labelled cells were rarely found in the anterior splanchnic mesoderm, where Tbx1, which is known to also be expressed in the SHF^38^,^39^, was distributed (Fig. 3g).

### *Sfrp5-YFP*-expressing ES cells differentiate into cardiac components

We next tested whether *Sfrp5*-*venusYFP*-expressing embryonic stem (ES) cells could differentiate into cardiac components, such as endothelial, smooth muscle and myocardial cells. On day 2, the peak of *YFP* expression was found to be ahead of those of other differentiation markers (Supplementary Fig. 8). On day 8, CD31-, α-smooth muscle actin- and TnT-positive cells were found in the cell mass (Supplementary Fig. 8), suggesting that *Sfrp5* could also serve as a marker gene of precursor cells for cardiac components in an *in vitro* differentiation system.

### Sfrps may not be involved in cardiac differentiation

**Figure 4:**
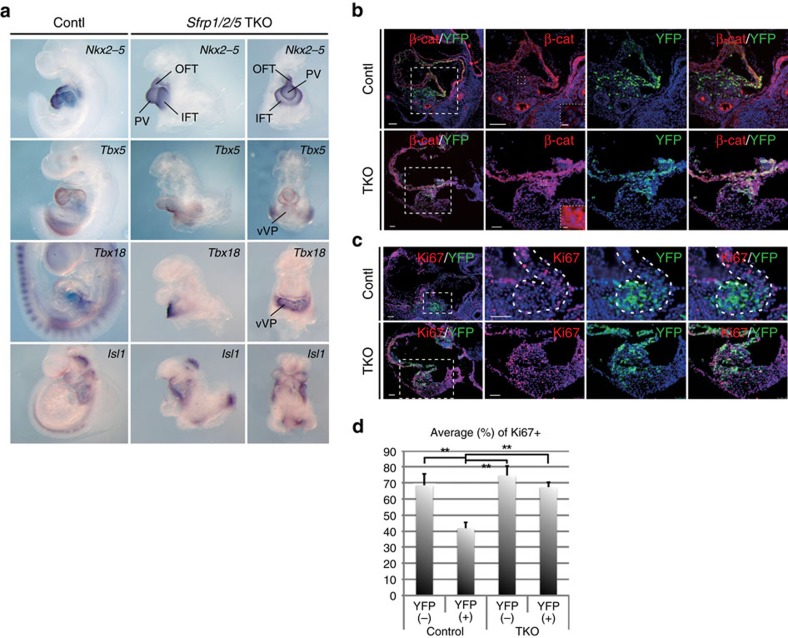
*Sfrp1/2/5* TKO embryos exhibit hyperplasia of the IFT accompanied by activated canonical Wnt signalling. (**a**) ISH of control and TKO embryos with probes for *Nkx2-5*, *Tbx5*, *Tbx18* and *Isl1* genes. PV: primitive ventricle. (**b**,**c**) Double immunohistochemistry using anti-β-catenin (**b**) or anti-Ki67 (**c**) antibodies in control and TKO embryos. Rectangles from the left end panels are magnified in the right panels. Further magnified photos, located in the right bottom window, show that β-catenin accumulated in YFP-expressing cells in the ventral venous pole in TKO embryos. (**d**) Percentage of Ki67 positive (+) cells in *Sfrp5*-*YFP*-expressing (+) or negative (−) cells in control and TKO embryos (*n*=3). Data are presented as mean±s.d.; **P<0.01, as compared with control samples. Scale bars=50 μm (5 μm in dotted rectangles).

Our results imply that *Sfrp5* is expressed and retained in cardiac progenitors but lost upon their differentiation, indicating that the loss of *Sfrp5* might enhance the differentiation of these cardiac progenitors. To test this possibility, we generated *Sfrp1/2/5* TKO mice. As reported previously, only TKO mice show severe growth retardation due to the compensatory functions of *Sfrp1* and *Sfrp2* (ref. 40). We focussed our analysis on the cardiac phenotype and analysed several lineage markers via *in situ* hybridization (ISH) at E9.5, the latest stage at which TKO embryos can be collected. Similar to controls, *Nkx2-5* was present in the heart tube but not in the venous pole in TKO embryos (Fig. 4a). This indicates that the differentiation of cardiomyocytes occurred normally until this stage. The expression of *Tbx5*, *Isl1* and *Tbx18* was normal (Fig. 4a), suggesting that formation of the FHF, SHF and SV was not affected. These observations suggest that *Sfrp*s may not affect differentiation.

### Sfrps prevent proliferation by inhibiting canonical Wnt signalling

TKO embryos appeared to have an enlarged IFT, as compared with the OFT. To determine whether Sfrps might regulate the proliferation of cardiac progenitors, we next assessed whether Wnt/β-catenin signalling and progenitor cell proliferation in the venous pole were affected in TKO embryos. In control embryos, β-catenin accumulated in the ventricles and atria, but less β-catenin was observed in YFP-positive cardiac progenitor cells in the ventral venous pole (Fig. 4b). In TKO embryos, however, β-catenin accumulated even in YFP-positive cardiac progenitor cells (Fig. 4b). In control mice, Ki67 was more highly expressed in YFP-negative cells than in YFP-positive cells, suggesting that proliferation may be inhibited in *Sfrp5*-expressing cardiac progenitors (Fig. 4c,d). In contrast, the percentage of Ki67-positive cells in TKO embryos increased in YFP-positive cells, as compared with the percentage in control samples (Fig. 4c,d). These results indicate that Sfrps prevent the proliferation of cardiac progenitors by inhibiting canonical Wnt signalling.

## Discussion

Based on our findings, we propose a model for the contribution of *Sfrp5*-expressing cardiac precursors to heart development, except for the RV (Supplementary Fig. 9). We found that *Sfrp5*-expressing cells at E7.5 contributed not only to the OFT, LV and atria and also to the SV by fate mapping experiments using *Sfrp5-Cre* and -*ERT2Cre* mice. This observation gives rise to the possibility that the FHF lineage and the SV might share a common origin. This idea has been implied previously by the expression and lineage-tracing experiments of *Hcn4*, because *Hcn4-*expressing cells ultimately yield most of the LV, portions of the atria and the cardiac conduction system^30^,^41^,^42^,^43^,^44^. We found that the expression of *Sfrp5* rarely overlapped with that of *Hcn4*, while cells weakly expressing YFP in the PV and IFT at E8.5 largely overlapped with *Hcn4*. The expression of *Sfrp5* also largely overlapped with that of *Tbx5* at E7.5, and early labelling of *Tbx5*-expressing cells at E6.5–E7.5 are reported to mark cells that are predominantly restricted to the LV and atria^36^,^37^, suggesting that *Sfrp5*-expressing cells include progenitors for the FHF. Moreover, *Sfrp5* was continuously expressed in cells giving rise to the SV, suggesting that cells that transiently express *Sfrp5* contribute to the FHF. *Sfrp5*-expressing cells start to express *Tbx18* at around E8.0, and forced expression of *Tbx18* reportedly promotes differentiation of cells into the SV, including the conduction system^45^, and represses a subset of myocardial genes^46^. Thus *Sfrp5*-expressing cells could maintain the potential of cells to differentiate into both the SV and FHF until *Tbx18* expression initiates.

Alternatively, the early *Sfrp5*-expressing progenitor pool might already be heterogeneous in the commitment to each differentiation program. Since *Tbx5*-expressing cells at E6.5–E7.5 would not contribute to the OFT (personal communication from Dr Yashiro), a precise comparison of expression domains and lineages of *Sfrp5* and *Tbx5* may discriminate *Sfrp5-*expressing progenitors that contribute to the OFT from those that contribute to other regions in the cardiac crescent. However, the expression domain of *Sfrp5* determined by ISH could be more restricted than it actually is at early stages, given the possibility of Cre recombination occurring in *Sfrp5*-expressing progenitors at levels undetectable by ISH. Thus the timing for cell fate determination and the potential for *Sfrp5*-expressing progenitor cells to differentiate should be addressed in future.

**Figure 5:**
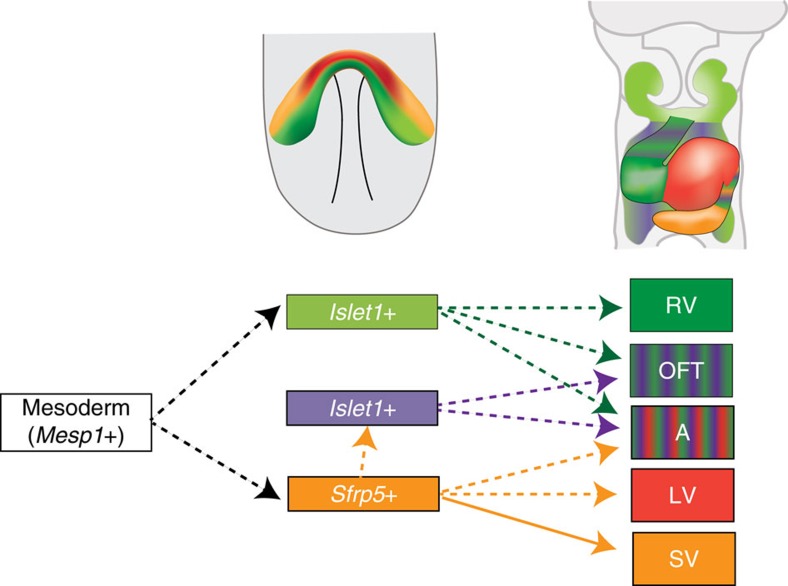
Model for the contribution of *Sfrp5*-expressing cardiac precursors to heart development. Upper panels show the expression area of *Sfrp5* (orange) and *Islet1* (light green) and the distribution of descendants of *Sfrp5-*expressing (red) and *Islet1-*expressing (green) cells at E7.5 (right) and E9.5 (left). The lower panel shows the lineage contribution to each part of the heart. Mesodermal cells, which transiently express Mesp1, may become cardiac precursor cells expressing *Islet1* (light green) or *Sfrp5* (orange). A portion of *Sfrp5*-expressing cells later begins to express *Islet1* (purple). Transient *Islet1-*expressing cells that do not express *Sfrp5* contribute to the RV, OFT and atrium (green), and cells that express *Sfrp5* contribute to the OFT and atrium (purple) but not to the RV. Cells that transiently express *Sfrp5* contribute to the LV, atrium and OFT (orange), and cells that continuously express *Sfrp5* form the SV.

Another observation was that a portion of *Sfrp5*-expressing cells later expressed *Isl1* and contributed to the OFT and atria (Fig. 5). This suggests that the *Isl1-*expressing SHF progenitor population has at least two distinct origins, one of which is derived from *Sfrp5*-expressing cells. We also found that *Tbx1* is expressed in the anterior splanchnic mesoderm in which descendants of *Sfrp5*-expressing cells did not appear, indicating that *Tbx1* could mark the subpopulation of *Isl1-*expressing cells in the SHF. Moreover, lineages of *Sfrp5*-expressing cells did not contribute to the RV. It was previously reported that mesodermal cells of the first branchial arch are involved in RV formation and that *Tbx1* is expressed in the first branchial arch and descendants of *Tbx1-*expressing cells contribute to the RV^38^,^39^,^47^,^48^,^49^. These findings suggest a distinct origin of the RV relative to that of the other chambers. Given that septated ventricles have been evolutionally acquired to separate systemic and pulmonary circulation^50^, our results provide new insight into the arrangement of cardiac progenitors before the evolutionary emergence of the RV.

The role of Wnt signalling in cardiac development has been extensively reported^17^. The hyperactivation of Wnt/β-catenin signalling in cardiac precursors leads to enhanced proliferation and inhibition of further differentiation^51^,^52^. In our study, increased proliferation was observed in TKO embryos, but differentiation into primitive cardiomyocytes was not impaired. We speculate that artificial activation and lack of inhibition of Wnt/β-catenin signalling may result in different outcomes during myocardial differentiation, and a primary role of Wnt/β-catenin signalling in this context may be the regulation of proliferation rather than differentiation. Since *Sfrp1/2/5* has been reported to regulate planar cell polarity pathways through the non-canonical Wnt pathway^40^, tissue- or cell-specific functions of Sfrps may exist. Further understanding of the molecular basis of cardiac progenitor differentiation into various types of cardiomyocytes could provide new therapeutic insights for regenerative medicine.

## Methods

### Mice

To generate *Sfrp5*-*Cre recombinase* (*Cre*) and *Sfrp5*-*modified ERT2 conjugated with Cre* knock-in mouse lines, a strategy similar to that used to generate the *Sfrp5*-*venusYFP* mouse line was adopted^40^. Targeting vectors were constructed by using a cassette containing *Cre*- or *ERT2Cre-recombinase* genes with *frt-PGK-neomycin (neo)-bpA-frt*, which was ligated with KpnI-SmaI and SpeI-XbaI fragments for long and short homologous arms, respectively. A MC1-DTA-negative selection marker cassette was also added to the outside of the short homologous arm to enrich for homologous recombinants (Supplementary Fig. 3). For recombination of the targeting vector for *ERT2Cre*, the CRISPR-Cas9 system was used. A 5′-GGCCAGGAGTACGACTACTACGG-3′ sequence was inserted into the pX330 plasmid (Addgene) using the BbsI site and was then co-transfected with the targeting vector. Homologous recombination was confirmed by southern hybridization for the *Sfrp5*-*Cre* line, using XbaI-EcoRI fragments for the probe, and by PCR for the *Sfrp5*-*ERT2Cre* line, using the following primer set: Neo6: 5′-CGACCACCAAGCGAAACATC-3′ and SAcontRR9: 5′-CCCAGGGAAGGGAATTGTTCTAG-3′. Deletion of Neo by crossing with the *ROSA-Flp* mouse line was confirmed by PCR, using the following primer set: DeltaNeoF1: 5′-CCTACATGCGCCCACTAGCCGT-3′ and DeltaNeoR2: 5′-CCTAGCGACCCCAAGCATCC-3′. TKO embryos were obtained by crossing with *Sfrp1/2/5*; −/−, +/−, −/− or −/−, +/−, venus (v)/v^40^. *Isl1-Cre* mouse lines have been previously generated using gene-targeting techniques^7^. The *ROSA26R* mouse line was a gift from Dr Soriano^31^. These lines were maintained in a 129 and C57BL/6 mixed background. A *CAG-floxed-CAT-lyn-mRFP* mouse line was newly and *CAG-floxed-CAT-eGFP* mouse line was previously established using transgenic techniques using C3HeJ × C57BL/6J F1 hybrid mice^53^.

### Tamoxifen injection

Tamoxifen (Sigma) was injected peritoneally into 8–20-week-old pregnant female mice at a dosage of 35 mg kg^−1^ at E7.5–E9.5. Embryos were dissected on E10.5 or E13.5 and subjected to lacZ staining.

### Whole-mount double fluorescent ISH

Embryos were dissected between E7.5 and E9.5 and fixed with 4% paraformaldehyde in PBS for durations of 1 h to overnight. After dehydration using a methanol series, embryos were rehydrated with 0.1% Tween 20 in PBS (PBST), bleached with 6% H_2_O_2_ in PBS, treated with Protease K and then hybridized with digoxygenin (DIG)- and/or dinitrophenyl-labelled probes at 68 °C overnight. They were then washed with 5 × and 2 × SSC/50% formamide, PBST and blocking reagent for 1 h. For detection of DIG-labelled probes, embryos were incubated with anti-DIG antibodies and detected using a TSA-Alexa555 detection system according to the manufacturer's instructions. For detection of dinitrophenyl, TSA-Alexa488 was used. Embryos were analysed by confocal laser microscopy (Leica LSM 5 PASCAL system).

### Immunohistochemistry and LacZ-staining

Embryos were embedded with optimum cutting temperature compound after fixation and were sectioned at 10–14 μm increments. After washing with PBS and 1 h of preincubation with 1% BSA in PBS for blocking, sections were incubated with 500 × diluted primary antibodies (anti-GFP: Nakalai GF090R or Abcam ab290; anti-Tbx1: Santa Cruz sc-17874; anti-Tbx18: Santa Cruz SC17869; anti-Wt1: Abcam ab10670; anti-Nkx2-5: Abcam ab35842; anti-TnT: Thermo MS-295-P0; anti-Hcn4: Alomone Labs APC052; anti-RFP: MBL PM005; anti-β-catenin: Millipore 05-665; anti-Ki67: Abcam ab15580) at 4 °C overnight. The sections were then incubated with 500 × diluted secondary antibodies conjugated with Alexa Fluor 488 or 555 (Molecular Probes). Nuclei were counterstained with 4,6-diamidino-2-phenylindole. For LacZ-staining, frozen sections were incubated with X-gal solution at room temperature overnight after washing with PBS.

### Cardiac induction of ES cells

ES cells were isolated from blastocysts of *Sfrp5-venusYFP* mice and maintained in Leukemia Inhibitory Factor-containing (1,000 U ml^−1^) ES culture medium (DMEM with 10% fetal bovine serum, 1 × Non-Essential Amino Acids Solution, 1 mM sodium pyruvate, and 10^−4^ M 2-mercaptoethanol). For cardiac induction, embryoid bodies, which were formed by the hanging-drop culture method for 48 h, were plated in 2-well chamber slides or 98-well plates (Nunc) and cultured for 8 days in ES culture medium without Leukemia Inhibitory Factor.

### Real-time PCR

Total RNA was isolated using TRIzol (Invitrogen). Reverse transcription was performed with the ReverTra Ace qPCR RT Kit (TOYOBO). Real-time PCR was conducted using SYBR Premix Ex Taq II (Takara Bio Inc. or Kapa Biosystems Inc.). Intensities of PCR products were measured and analysed using Mini-Opticon (Bio-Rad). The following primers were used: *YFP*: forward 5′-CCTGGTCGAG CTGGACGGCGAC-3′ and reverse 5′-TCACGAACTCCAGCAGGACCATG-3′, *Nkx2-5*: forward 5′-GCTACAAGTGCAAGCGACAG-3′ and reverse 5′-GGGTAGGCGTTGTAGCCATA-3′, *β-MHC*: forward 5′-TCCACGGGGAAGAGCATCCAT-3′ and reverse 5′-CAGACTCTGGAGGCTCTTCACT, *Hcn4:* forward 5′-CTCAACTCAGGCGTCTTCAAC-3′ and reverse 5′-CCACCGAAGTAGTAGCAGCAG-3′, and *G3pdh*: forward 5′-ACCACAGTCCATGCCATCAC-3′ and reverse 5′-TCCACCACCCTGTTGCTGTA -3′. Amplification conditions were 5 s at 95 °C, 20 s at 60 °C and 15 s at 72 °C for 49 cycles. *G3pdh* was amplified as an internal control.

### Statistical analysis

A parametric multiple comparison test, one-way analysis of variance with Tukey–Kramer *post hoc* analysis, was mainly used (unless otherwise indicated) when Bartlett's test (*P*<0.05) showed homogeneity of variances with the Ekuseru–Toukei 2012 software (Social Survey Research Information Co., Ltd.). All data are expressed as mean±s.d. *P*<0.05 was considered statistically significant.

### Data availability

The authors declare that all data supporting the findings of this study are available within the paper and its Supplementary Information files or available on reasonable request from the corresponding authors.

## Additional information

**How to cite this article:** Fujii, M. *et al*. *Sfrp5* identifies murine cardiac progenitors for all myocardial structures except for the right ventricle. *Nat. Commun.* **8,** 14664 doi: 10.1038/ncomms14664 (2017).

**Publisher's note:** Springer Nature remains neutral with regard to jurisdictional claims in published maps and institutional affiliations.

## Supplementary Material

Supplementary Information Supplementary Figures, Supplementary reference
