# Supplementary material for: Sfrp5 identifies murine cardiac progenitors for all myocardial structures except for the right ventricle
Source: Nat Commun. 2017 Mar 13;8:14664. doi: 10.1038/ncomms14664 (PMC5355806; doi:10.1038/ncomms14664)
Supplement: Supplementary Information — Supplementary Figures, Supplementary reference [file ncomms14664-s1.pdf]

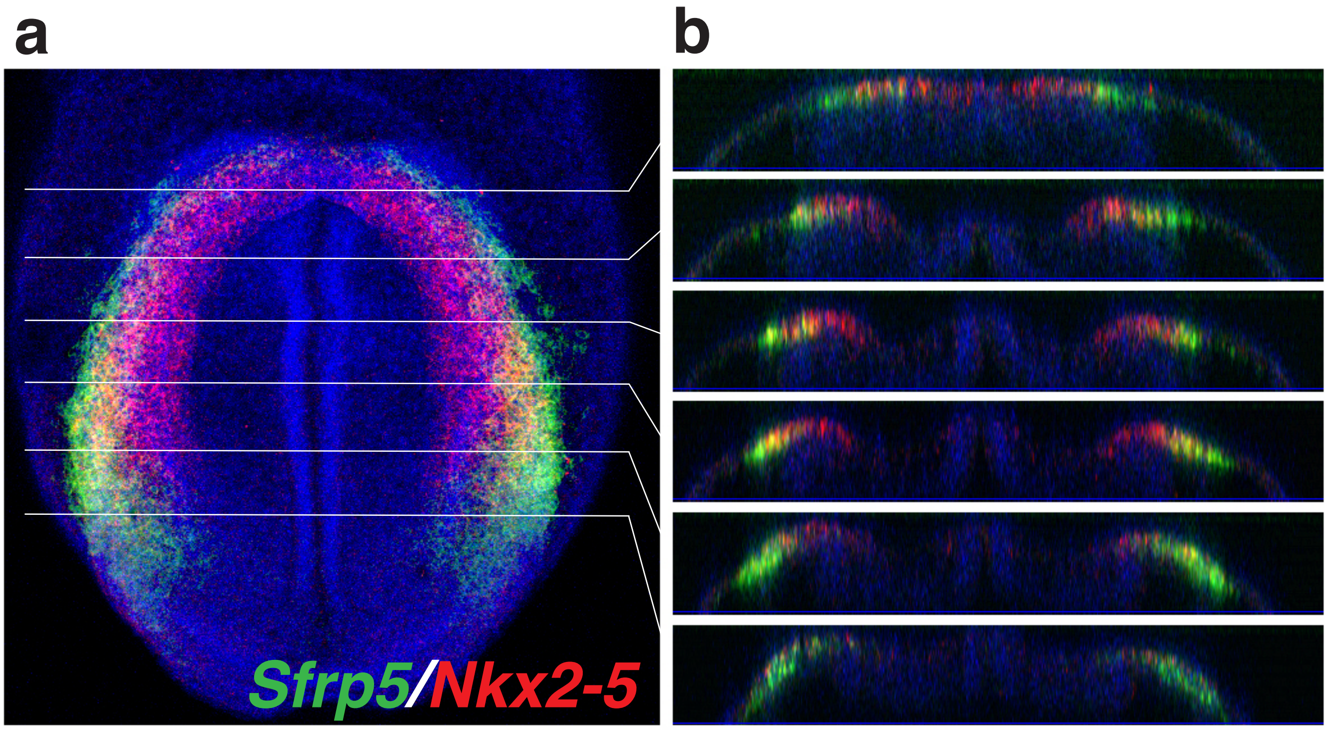

Supplementary Figure 1: *Sfrp5* is expressed in lateral regions of the cardiac crescent. a: Representative view of whole mount double fluorescent *in situ* hybridization using *Sfrp5* (green) and *Nkx2-5* (red) probes at E9.5 by scanning with a laser scanning microscope (LSM). b: Re-constituted transverse sections at each line in panel a.

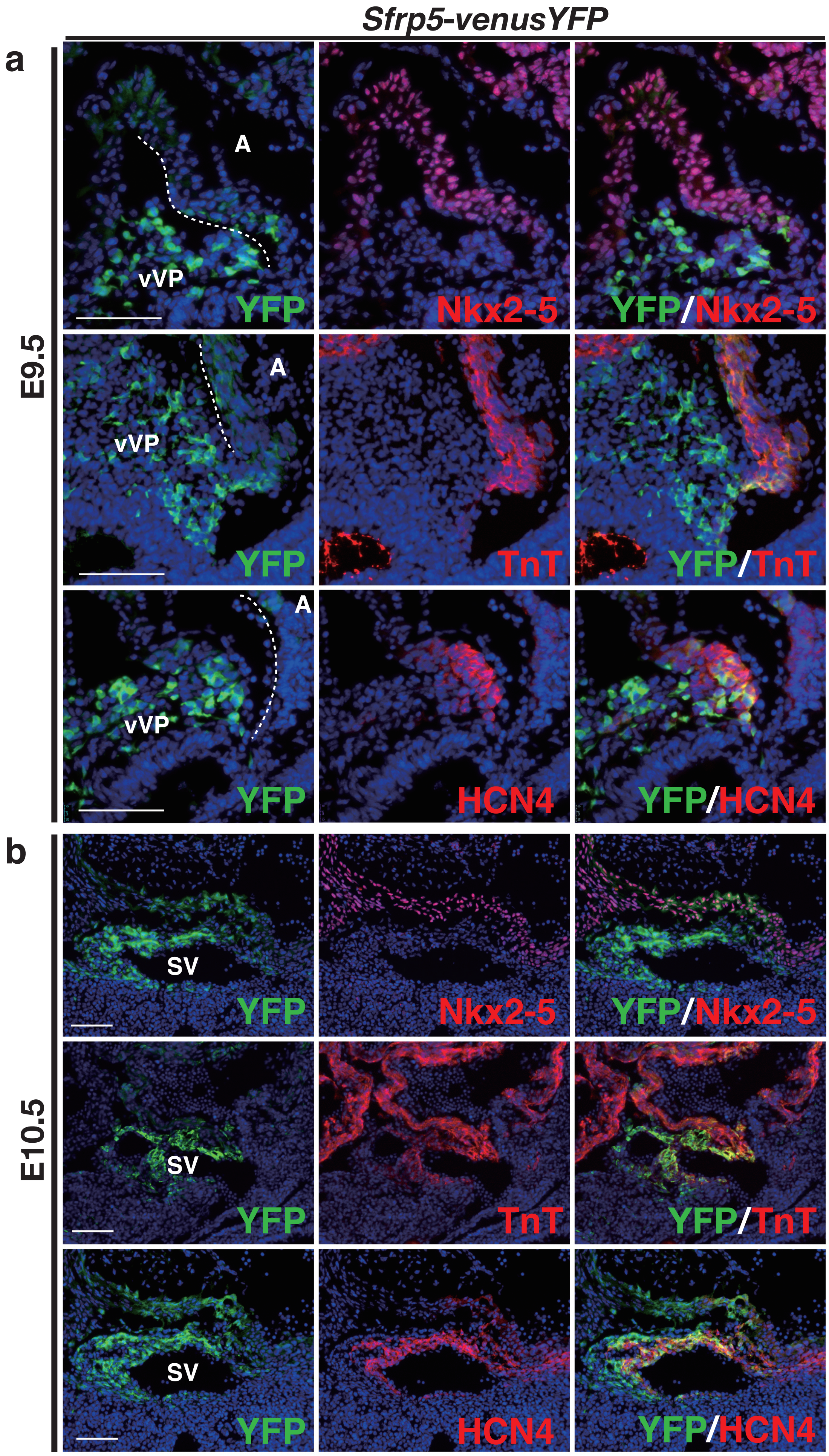

Supplementary Figure 2: *Sfrp5*-expressing cells in the ventral venous pole differentiate into Hcn4- and TnT-positive, Nkx2-5-negative, cardiomyocytes at E10.5. a, b: Double fluorescent immunohistochemistry using anti-GFP (green) with anti-Nkx2.5, anti-TnT, or anti-Hcn4 antibodies (red) in *Sfrp5-venusYFP* embryos at E9.5 (**a**) and E10.5 (**b**). Nkx2-5, TnT, and Hcn4 were not detected in *Sfrp5-venusYFP*-expressing mesenchymal cells in the ventral venous pole (vVP) at E9.5. TnT and Hcn4, but not Nkx2-5, were found in the forming sinus venosus (SV) at E10.5. Scale bars=50 µm.

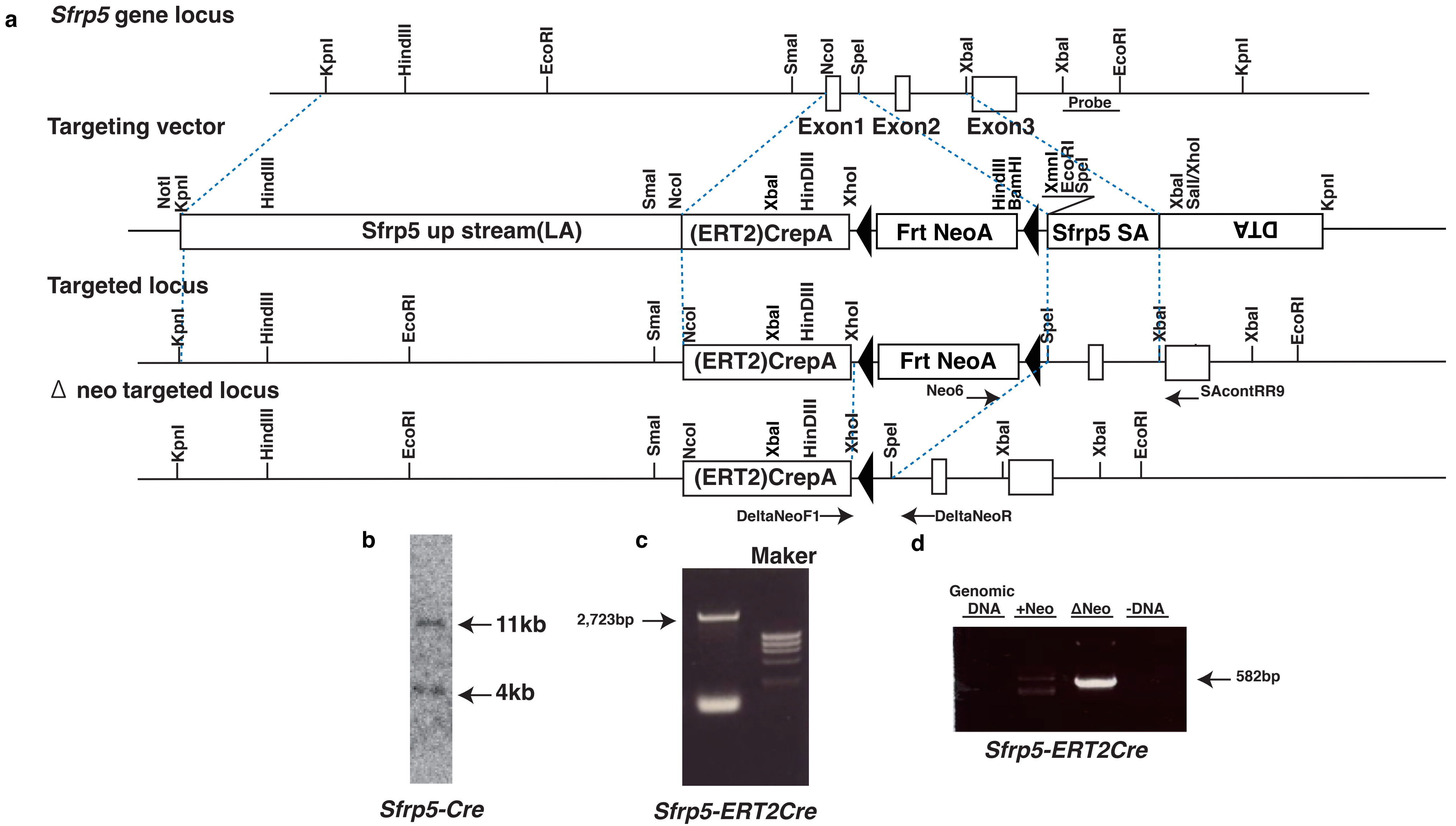

Supplementary Figure 3: Targeting strategy for *Sfrp5* gene locus for generation of *Cre* or *ERT2Cre* KI mouse lines. a: Schematic drawing of targeting straegy. *Sfrp5* gene locus is shown at the top. Genomic fragments for long arm and short arm (LA and SA) were inserted in either the *Cre-* or *ERT2-Cre-Frt-neo* cassette and the *Diphtheria toxin-A* (*DTA*) cassette to generate targeting vectors. After targeting events, almost all regions of Exon1 were replaced in *Cre-* and *ERT2-Cre-Frt-neo* cassettes (targeted locus). *Frt-neo* was then removed by crossing with *Rosa-Flp* mice (Δneo targeted locus). **b:** Southern blotting of the EcoRI digested genome from the *Sfrp5-Cre* targeted ES line, using the XbaI-EcoRI fragment as a probe. The expected fragment (4kb) after the targeting event was detected. **c:** Detection of the targeting event for the *Sfrp5-ERT2Cre* targeted ES line by PCR was conducted using the probe set, located on the outside of the short arms, Neo6 and SAcontRR9. The expected fragment (2,723 bp) was amplified. **d:** Confirmation of neo cassette deletion by PCR was conducted using the probe set, located on the outside of the neo cassette, DeltaNeoF1 and DeltaNeoR2. The expected fragment (582 bp) was amplified using genomic DNA from delta (Δ) neo mice, but not from wild type or neo cassette containing (+) mice, or those without (-) genomic DNA.

a

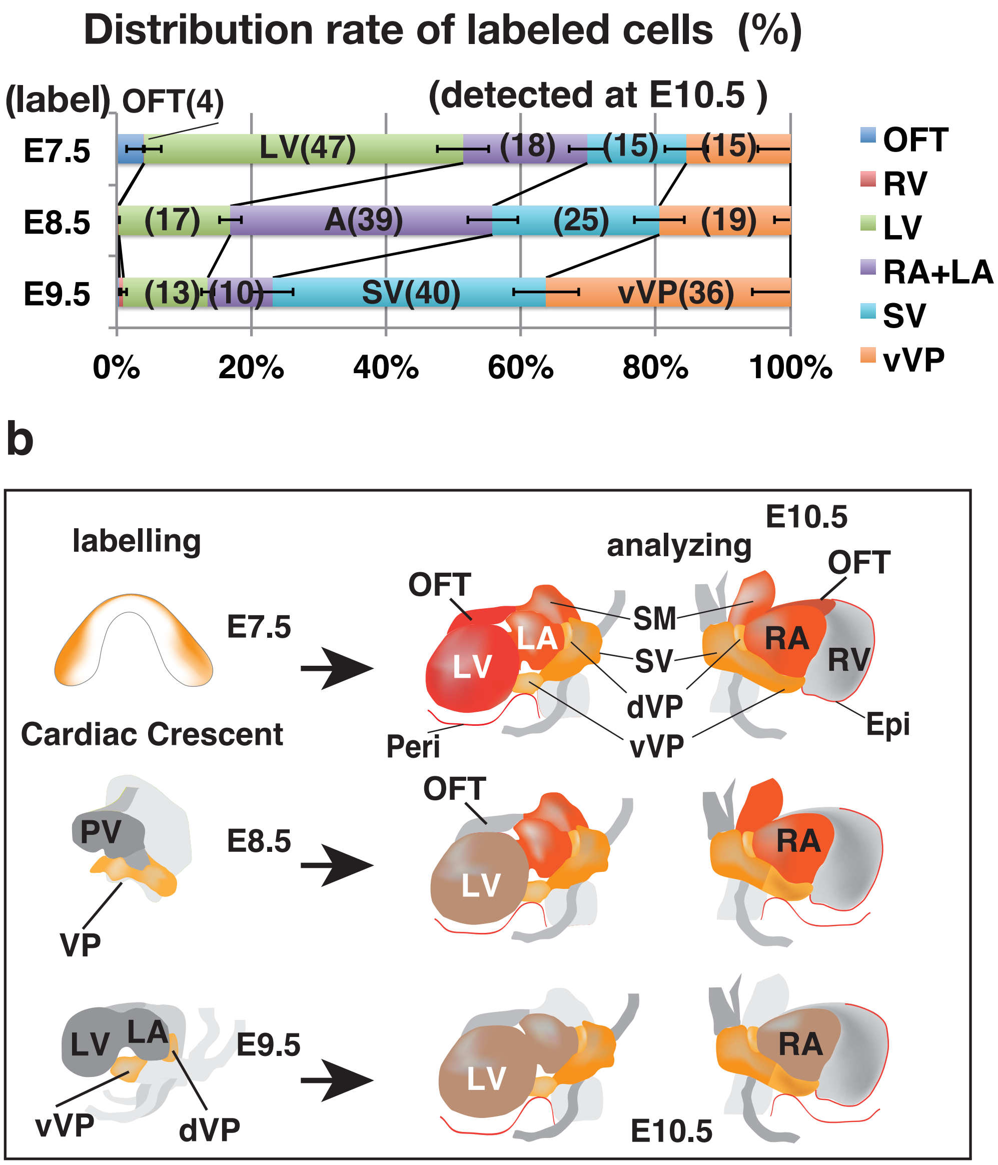

Supplementary Figure 4: *Sfrp5*-expressing cells supply cardiomyocytes in the inflow tract until E10.5. a: Distribution rate (%) of cells at E10.5, labelled at E7.5 (n=5 in three injections), E8.5 (n=3 in two injections), or E9.5 (n=3 in one injection), in the OFT, LV, RV, LA and RA, sinus venous (SV), and ventral venous pole (vVP). The major contributing domains of labelled cells shifted from the LV (at E7.5) to the atria (at E8.5), and then to the SV and the VP (at E9.5). **b:** Schematic drawing for the expression of *Sfrp5* (orange) and the cardiac contribution of its lineages (red), when cells were labelled at E7.5, E8.5, or E9.5. These drawings were inspired by our own work and data in Figure 2 (1) .

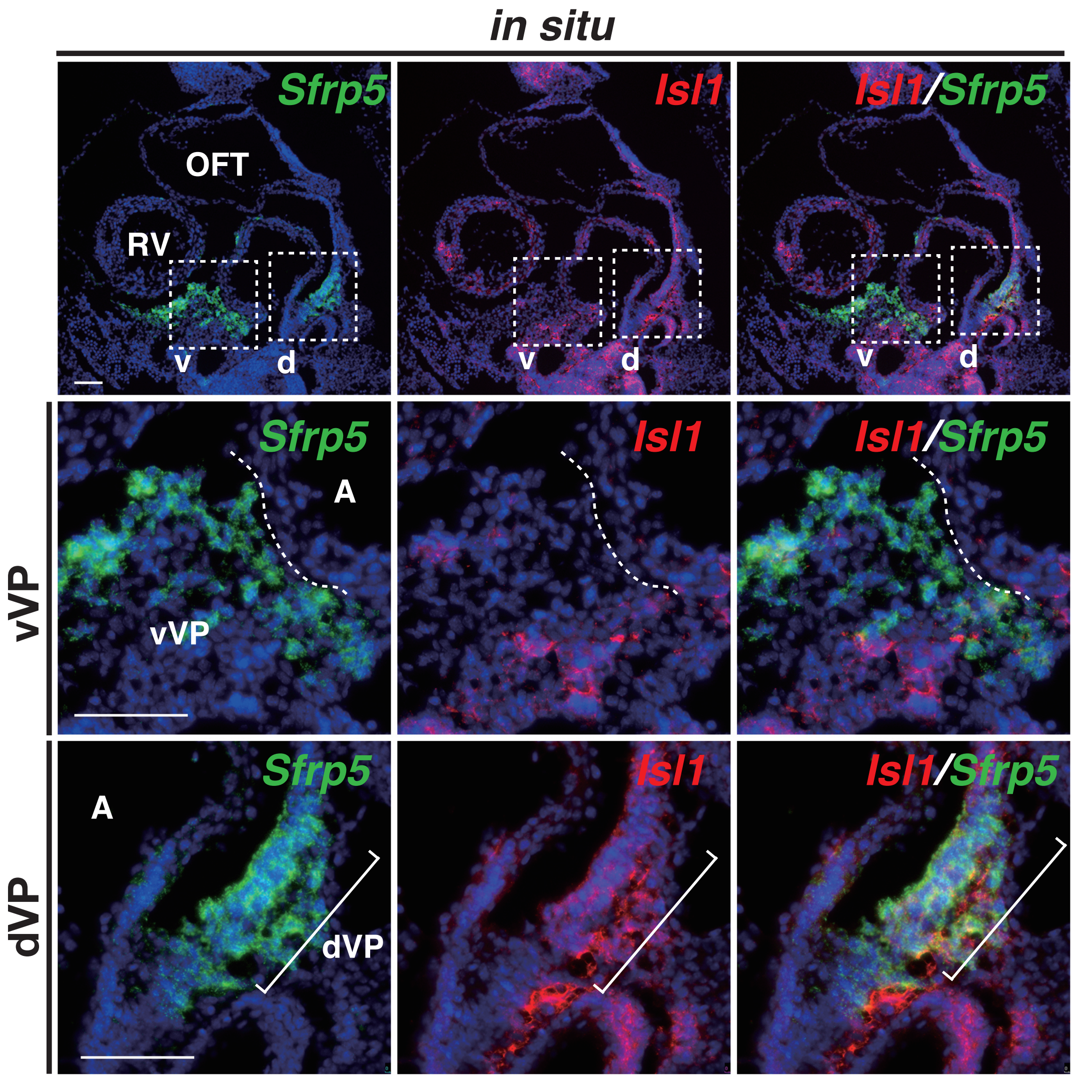

Supplementary Figure 5: *Sfrp5* is co-expressed with *Isl1* in the dorsal venous pole but not in the ventral venous pole. Double fluorescent in situ hybridization at E9.5 using *Sfrp5* and *Isl1* probes. Rectangles, indicating d and v in the upper panels, were magnified in lower panels to clearly show the dorsal and ventral venous poles (dVP and vVP). In the dVP, expression of *Sfrp5* was found in mesenchymal cells (from the left side to the white dotted line), while weak expression of *Isl1* was detected in a few mesenchymal cells. *Isl1* expression is found predominantly in the dVP, while co-expression with *Sfrp5* is detected in some sections (white bracket). Scale bars=50 µm. OFT: outflow tract; RV: right ventricle; A: atrium.

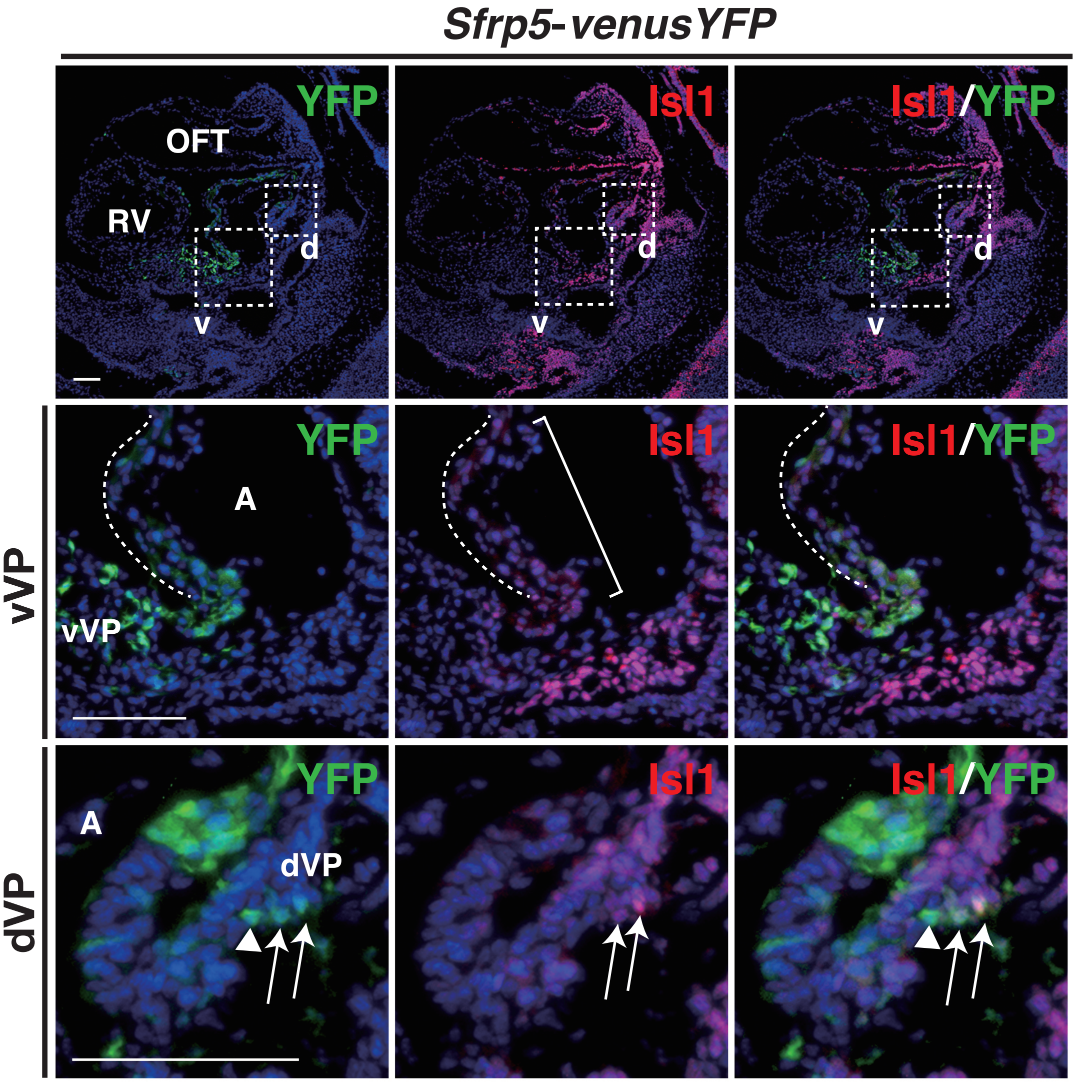

Supplementary Figure 6: Co-distribution of Sfrp5 with Isl1 was detected in the dorsal venous pole but not in the ventral venous pole. Double fluorescent immunohistochemistry using anti-GFP and anti-Isl1 antibodies in *Sfrp5-veneusYFP* KI embryos. In the ventral venous pole, YFP was strongly expressed in mesenchymal cells (from the left side to the white dotted line), while distribution of Isl1 was strong in the splanchnic mesoderm and weak in the atria (white bracket). In the dorsal venous pole, the intensity of YFP was significantly lower than that in the ventral venous pole. Distribution of enhanced YFP (arrowhead) and co-distribution of YFP with Isl1 (arrows) were detected in some cells of the dorsal venous pole in the lowest panels. Scale bars=50 μm. OFT: outflow tract; RV: right ventricle; A: atria.

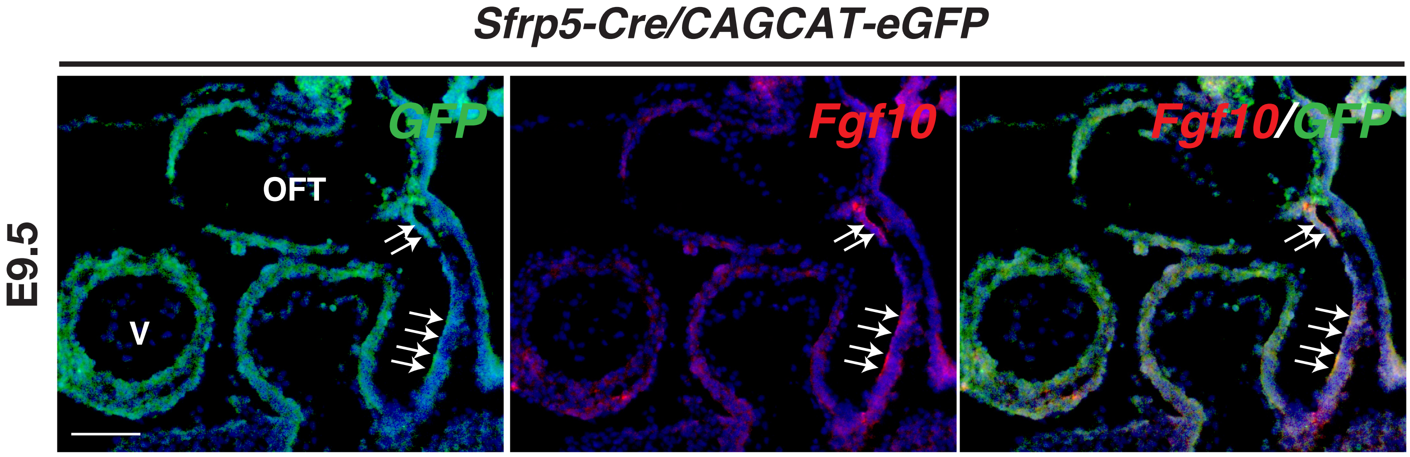

Supplementary Figure 7: *Fgf10* is expressed in *Sfrp5*-expressing cells of the dorsal venosus pole. Double fluorescent *in situ* hybridization at E9.5 using *GFP* and *Fgf10* probes in the *Sfrp5-Cre/CAG-loxp-CAT-eGFP* embryo. In the dorsal venous pole, expression of *Fgf10* was found in the ventral side of splanchnic mesodermal cells (arrows) together with *Sfrp5*. Scale bars=100 µm. OFT: outflow tract; V: ventricle.

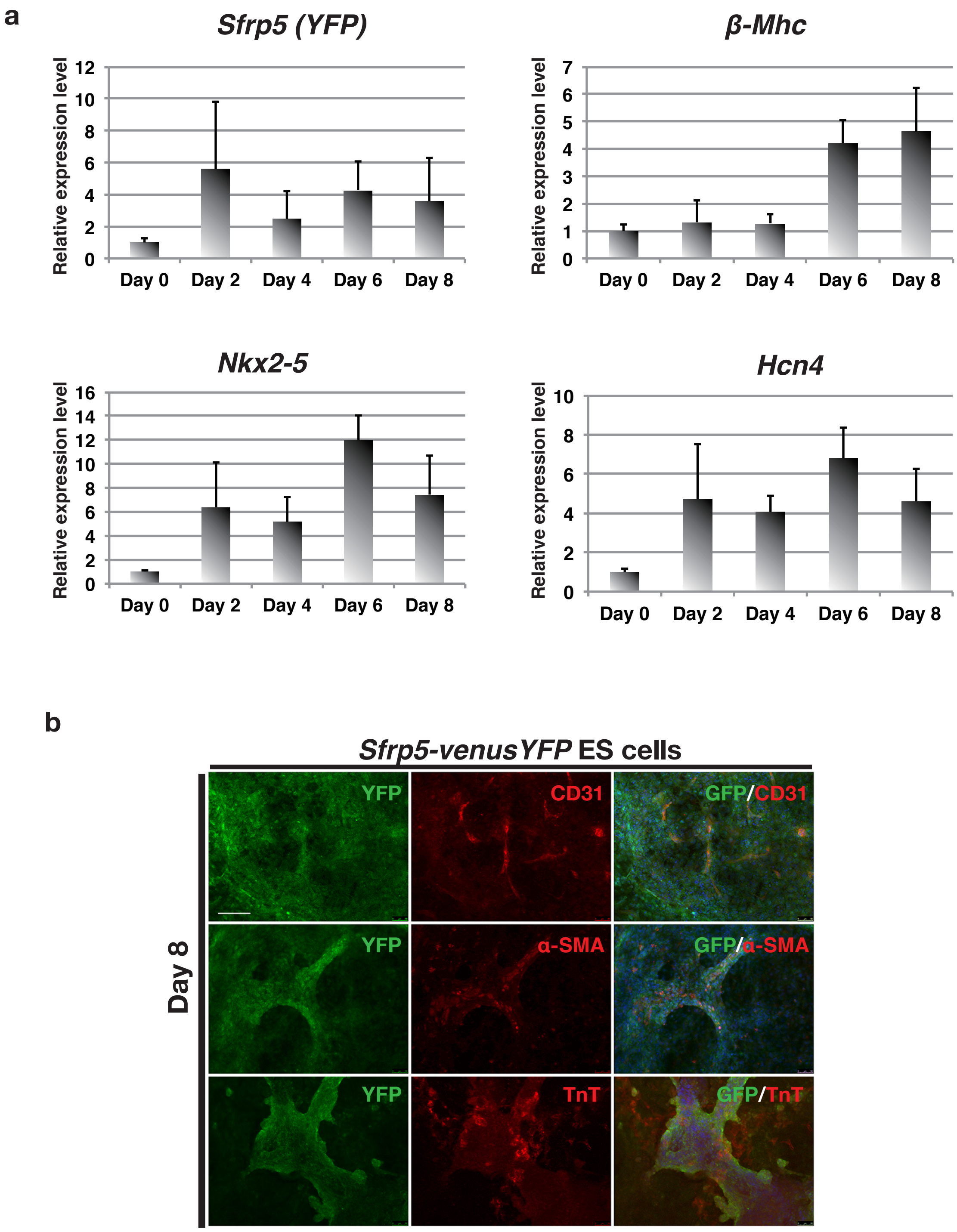

Supplementary Figure 8: *Sfrp5*-expressing ES cells differentiate into endocardial, smooth muscle, and myocardial cells. a: The quantitative RT-PCR analysis of *Sfrp5-venus YFP* KI ES cells on the indicated culture days after cardiac induction (n=5). **b:** Double fluorescent immunohistochemistry using anti-GFP with anti-CD31, anti-SMA, or anti-TNT antibodies in the differentiated cell mass derived from *Sfrp5-venus YFP* KI ES cells. Scale bars=100 μm.

Supplementary reference

1. Mommersteeg, M. T. et al. The sinus venosus progenitors separate and diversify from the first and second heart fields early in development. Cardiovascular research 87, 92-101 (2010).
